# Editorial for the Special Issue on Emerging Micro Manufacturing Technologies and Applications

**DOI:** 10.3390/mi14061248

**Published:** 2023-06-14

**Authors:** Nikolaos Tapoglou

**Affiliations:** Industrial Engineering and Management Department, International Hellenic University, 57001 Thessaloniki, Greece; ntapoglou@iem.ihu.gr

In recent years, the field of micromachining has gained a lot of traction owing to the drive towards lightweighting, electrification, and sustainability. Industrial sectors that have shown an increasing interest in micromachining include the medical, space, aerospace, and consumer electronics fields. Research in academia has focused on the experimental investigation of micromachining and additive manufacturing processes and in particular, on laser-based manufacturing technologies. In addition, numerical and finite element models have been developed to predict the performance of micromachined parts. Over the last few years, a series of manufacturing processes have emerged in the macro manufacturing sector that have shown great potential in the improvement of these processes; however, their use on the micro scale has not been thoroughly modeled and understood. Moreover, a series of processes developed to address challenges in micro-manufacturing have been emerging as a parallel thread. Accordingly, this Special Issue showcases nine original research papers and one state-of-the-art review in the fields of manufacturing micro-electronic devices, surface property modification, and additive manufacturing.

In particular, Hauschwitz et al. [1] presented a novel technique for the modification of the reflectivity and surface topography of tempered glass using an ultrashort, pulsed laser. By utilizing a dynamic beam shaping and a galvanometer scanning head, the laser beam was divided into a matrix of beamlets, allowing for the fast and flexible fabrication of a sub-wavelength ripple structure on the surface. The study showed that reflected intensity reduced by up to 75% while maintaining 90% of transparency.

Kluba et al. [2] presented a novel type of substrate for a Silicon on Insulator wafer, which contained a patterned, buried oxide layer that can simplify the fabrication of MEMS devices with complex geometry and added functionality. The authors successfully demonstrated the application of the cavity-BOX SOI substrate in the fabrication of a deep brain stimulation (DBS) demonstrator with a length of 18 mm and a diameter of 1.39 mm.

Li et al. [3] developed a 2D model of the molten flow behavior during laser polishing, demonstrating the complex evolution of melt hydrodynamics involving heat conduction, thermal convection, thermal radiation, melting, and solidification. The model developed was able to predict that the morphological evolution of different surfaces from rough to smooth in laser polishing could guide the optimization of polishing parameters such as laser power and scanning speed. The experimental results showed a good correlation between the experimental and simulated results with an error ranging from 8.3 to 14.3%.

Cao et al. [4] investigated the performance of a Ag-8.5Au-3.5Pd alloy wire after cold deformation and annealing, using SEM, strength, and resistivity testing. The experimental campaign showed that the strength of the wire increases with the increase in the deformation rate, and the resistivity decreases with the increase in the annealing temperature. The mechanical performance of the alloy wire was improved at an annealing temperature of 500 °C. The surface quality is high when the tension range is 2.5–3.0 g. However, when the annealing temperature increased to 550 °C, the grain size growth led to a decrease in the mechanical performance.

Zhang et al. [5] presented a modified Bosch etching process to create silicon nanowires with a controllable density and high aspect ratios. The use of Au nanoparticles as a hard mask resulted in an anti-reflection property with a reflectance value below 2% in a broad light wave range and a near-unity reflectance below 3% in the range from 220 to 2600 nm. Additionally, the nanowire array demonstrated super-hydrophobic behavior without any hydrophobic chemical treatment.

Kim et al. [6] investigated the deposition of low-hydrogen-containing amorphous silicon (a-Si) using a plasma system with multi-split electrodes. Based on their experimental research, they concluded that increasing the RF power of the plasma led to decreased hydrogen content in the deposited film and a decrease in impurities such as carbon and oxygen. When crystalized under a UV lamp, a-Si exhibited improved crystallinity, confirmed by Raman spectroscopy and HR-TEM.

Kumamoto et al. [7] introduced a technique for fabricating metal structures that could be deformed from two-dimensional to three-dimensional shapes in response to hydrodynamic forces using photolithography and electroforming. The resulting structures had an average film thickness of 12.9 µm, a hardness of 600 HV, and a slit width of 7.9 µm.

Maddu et al. [8] evaluated the use of a bronze electrode for depositing copper material on titanium alloy using electrical discharge machining (EDM). The input parameters of current, Ton, Toff, and preheating substrate temperature were optimized using the Taguchi experimental design and the TOPSIS technique. The study found that input parameters of current 8 Amp, Ton 440 µs, Toff 200 µs, temperature 300 °C, and a quenching medium of castor oil provided the optimum response of an MDR of 0.00506 g/min, EWR of 0.00462 g/min, CT of 40.2 µm, and SCD of 19.4 × 10^7^ µm^2^.

Yakin et al. [9] aimed to optimize Fused Deposition Modeling (FDM) printing parameters for ABS and Nylon to improve fatigue resistance. The methodology involved experimental study and finite element analysis. The results showed that Nylon performed better than ABS, and the ‘tri-hexagon’ structure with a nozzle diameter of 0.2 mm resulted in the highest fatigue life for both materials.

Finally, Zhou et al. [10] discussed the history and development of bonding wires in microelectronic packaging, specifically comparing the properties and applications of Au, Cu, and Ag bonding wires. The paper highlighted the benefits and challenges of each material and suggested future research focuses on understanding the bonding mechanism, developing environmentally friendly surface treatment technology, exploring multi-component microalloy doping processes, and optimizing wire loop profiles for improved reliability.
